# Questions, as to the Velocity of the Blood’s Movements

**Published:** 1862-03

**Authors:** 


### QUESTIONS, AS TO THE VELOCITY OF THE BLOOD'S MOVEMENTS.

1st—Is the teaching that the heart sends into the aorta 3 oz of blood per second, and that this blood moves 12 inches in the same length of time, consistent with the known size of that vessel?

2nd—Considering the aorta always filled with blood, (*which must be the case,*) can the blood move farther per second than the 3 oz of blood would fill the cavity of the aorta in the quiescent state?

This, if the diameter of the aorta is 1 inch, would be near 7 inches. If 15 inches, would be little more than 3 inches.

3rd—If the 3 oz of blood received each second, fill 6 inches of the cavity of the aorta, and move 12 inches in the same length of time, would it not get 6 inches clear in advance of the following 3 oz of the next second, or leave the artery but partly full?

4th—Are the combined cavities of the capillaries, arising from any given artery, four hundred times as large as the artery, which must be the case,) if the (as we are taught) blood moves 12 inches per second in the arteries, and moves 3 inches in the capillaries, in the same length of time?

5th—Make the aorta four hundred times as large as it is, and would a man be "*big enough to hold it?*"

6th—What is the diameter of the ascending aorta?

Will some one please answer?

Respectfully, R. H. C.

